# Impact of adjuvants on the biophysical and functional characteristics of HIV vaccine-elicited antibodies in humans

**DOI:** 10.1038/s41541-022-00514-9

**Published:** 2022-08-04

**Authors:** Shiwei Xu, Margaret C. Carpenter, Rachel L. Spreng, Scott D. Neidich, Sharanya Sarkar, DeAnna Tenney, Derrick Goodman, Sheetal Sawant, Shalini Jha, Brooke Dunn, M. Juliana McElrath, Valerie Bekker, Sarah V. Mudrak, Robin Flinko, George K. Lewis, Guido Ferrari, Georgia D. Tomaras, Xiaoying Shen, Margaret E. Ackerman

**Affiliations:** 1 Quantitative Biomedical Science Program, Dartmouth College, Hanover, NH USA; 2 Thayer School of Engineering, Dartmouth College, Hanover, NH USA; 3 Department of Surgery, Duke University School of Medicine, Durham, NC USA; 4 Duke Human Vaccine Institute, Duke University Medical Center, Durham, NC USA; 5 Vaccine and Infectious Disease Division, Fred Hutchinson Cancer Research Center, Seattle, WA 98109 USA; 6 Departments of Laboratory Medicine and Medicine, University of Washington, Seattle, WA USA; 7 Division of Vaccine Research, The Institute of Human Virology, University of Maryland School of Medicine, Baltimore, MD USA

**Keywords:** Antibodies, HIV infections, Adjuvants

## Abstract

Adjuvants can alter the magnitude, characteristics, and persistence of the humoral response to protein vaccination. HIV vaccination might benefit from tailored adjuvant choice as raising a durable and protective response to vaccination has been exceptionally challenging. Analysis of trials of partially effective HIV vaccines have identified features of the immune response that correlate with decreased risk, including high titers of V1V2-binding IgG and IgG3 responses with low titers of V1V2-binding IgA responses and enhanced Fc effector functions, notably antibody-dependent cellular cytotoxicity (ADCC) and antibody-dependent cellular phagocytosis (ADCP). However, there has been limited opportunity to compare the effect of different adjuvants on these activities in humans. Here, samples from the AVEG015 study, a phase 1 trial in which participants (*n* = 112) were immunized with gp120_SF-2_ and one of six different adjuvants or combinations thereof were assessed for antibody titer, biophysical features, and diverse effector functions. Three adjuvants, MF59 + MTP-PE, SAF/2, and SAF/2 + MDP, increased the peak magnitude and durability of antigen-specific IgG3, IgA, FcγR-binding responses and ADCP activity, as compared to alum. While multiple adjuvants increased the titer of IgG, IgG3, and IgA responses, none consistently altered the balance of IgG to IgA or IgG3 to IgA. Linear regression analysis identified biophysical features including gp120-specific IgG and FcγR-binding responses that could predict functional activity, and network analysis identified coordinated aspects of the humoral response. These analyses reveal the ability of adjuvants to drive the character and function of the humoral response despite limitations of small sample size and immune variability in this human clinical trial.

## Introduction

Adjuvants play a major role in influencing the magnitude and characteristics of responses to protein vaccines. They have the capacity to elicit an elevated, broadened, and higher affinity antibody response^1–6^, and to drive engagement of innate immune receptors and generation of T-helper cell responses^7^. Adjuvants exert their action both by creating an antigen depot that exposes the antigen to the immune system for a prolonged period of time and enhancing interactions with antigen presenting cells^8–10^, as well as by activating the innate immune system by acting as ligands for pattern recognition receptors such as Toll Like Receptors (TLRs)^11^, which in turn result in the downstream activation of genes that code for chemokines, cytokines, and other costimulatory molecules^12^.

Despite their established importance in shaping the immune response, relatively few adjuvants have been approved for clinical use^13^, and selection among them is influenced by their safety profile, physicochemical characteristics and suitability for formulation, interactions with and effect on the vaccine immunogen, the intended immunization route, and the desired immune response, among other factors^7,14^. Comprehensive evaluation of the effect of adjuvants in humans has been somewhat limited by practical constraints, leaving a relative paucity of foundational comparative clinical data on which to base vaccine development decisions.

Perhaps there is no infectious disease vaccine that might benefit more from optimal adjuvant selection than HIV-1. While animal models have shown that antibodies and T cell responses can provide robust protection from infection^15–18^, there is little evidence of naturally acquired immunity^19^, and the first efficacy trials of a monoclonal antibody points to further barriers ahead^20^. These challenges to HIV vaccine development include the absence of an ideal animal model, the exceptional hyper-variability of HIV, and early establishment of a latent reservoir of infected cells^21–24^. Immunodominant epitopes are highly variable; conserved epitopes on envelope proteins are often masked by glycan, occluded within the envelope trimer, and sometimes only exposed after conformational changes^25–28^. As a result, potent and broad neutralizing antibodies against HIV-1 are rather rare and appear to have complex and slow developmental pathways^29^, further complicating the design of a suitable vaccine.

While each of these and other challenges have impeded induction of protective neutralizing antibody responses to HIV-1, antibodies with potent antiviral activity mediated by their ability to recruit innate immune effector cells and complement via their Fc domains have been found to be effective in providing protection against diverse pathogens^30–35^. Evidence supporting the potential relevance of antibody effector functions to vaccine-mediated protection from HIV infection has accumulated over decades of study in long-term non-progressor and elite controller cohorts, preclinical animal models, and now across multiple human HIV-1 vaccine efficacy trials^29,36–41^. In the well-known RV144 HIV vaccine trial, it was found that vaccination with a canarypox-protein HIV antigen (ALVAC–HIV and AIDSVAX HIV gp120, clade B and E proteins) formulated in alum elicited IgG antibodies against the first and second variable domains (V1V2) of Env that were not broadly neutralizing, but demonstrated antibody-dependent cell-mediated cytotoxicity (ADCC)^30^. These Env-specific IgG responses, in the absence of high IgA, were associated with reduced risk of infection^40,42^. This regimen also induced IgG3 antibodies with highly coordinated Fc-mediated effector functions that were associated with reduced risk of infection^43,44^.

In comparison, the HVTN 505 trial, which lacked overall vaccine efficacy (VE), immunization with a DNA/recombinant adenovirus 5 (rAd5) (no adjuvant) vaccine elicited low IgG3, low V1V2 IgG, but a high anti-gp120 IgA response in serum^45,46^. Follow-up analyses on the HVTN 505 trial revealed correlates of risk of infection – on one hand, elevated HIV-specific antibody binding to FcγRIIa, ADCP, and anti-Env IgG3 breadth correlated with reduced acquisition risk, while on the other hand, HIV-specific IgA modulated the association of Fc function with HIV-1 risk^41^. That these studies have shown divergence in outcomes that associate with specific antibody types demonstrates the potential importance of adjuvant selection to drive protective humoral responses. Both trials suggest that certain specificities of circulating IgA responses, which potentially interfere with the function of IgG antibodies^47^ should be avoided.

Further evidence of the potential significance of antibody activities beyond neutralization comes from preclinical studies in the non-human primate (NHP) model^48–54^. A few recent NHP studies have linked the choice of adjuvant both to the Fc effector function of the humoral response to vaccination and to protection against infection^55–58^. Om et al. evaluated a vaccinia virus prime and multimeric gp145 boost in the context of two different adjuvants, Army Liposome Formulation adsorbed to alum (ALFA) and alum alone, and found antibody-dependent neutrophil-mediated phagocytosis (ADNP), ADCC, ADCP, and antibody-dependent natural killer intracellular cytokine staining (NK ICS) responses to be elicited, but only the ALFA-adjuvanted regimen was observed to reduce the per-exposure risk of infection as compared to controls^55^. Vaccari et al. compared an ALVAC-SIV prime and gp120 boost adjuvanted with alum versus MF59, an oil-in-water emulsion adjuvant, and observed variations in antibody effector functions between the two adjuvant arms^56^. While the magnitude of most aspects of the humoral response was elevated by MF59, only alum was found to decrease the risk of SIVmac251 acquisition compared to unvaccinated controls^56^. Similarly, Kasturi et al. reported adjuvant-dependent differences in systemic versus mucosal antibody profiles and the association between non-neutralizing antibodies and protection from infection^57^. Whether in the context of a virus-like particle or recombinant protein, a nanoparticle adjuvant formulation that included TLR 4 and TLR7/8 ligands monophosphoryl lipid A (MPL) and R848 provided improved challenge resistance as compared to alum. Flow cytometric analysis of innate immune cells and blood provided linkages between these stimuli, antibody responses, and challenge resistance. Building on this result, a novel TLR 7/8 agonist has been reported to further improve titers and the longevity of humoral responses^58^.

Limited human immunogenicity data comparing adjuvants are available, particularly with respect to impacts on antibody isotypes and subclasses elicited by immunization. Additionally, immunogenicity profiles of adjuvants in animal models have often been compared pairwise. However, Francica et al. evaluated the induction, durability, and biophysical profiles of anti-HIV antibody responses in NHPs in the context of eight different adjuvant compositions^59^. Diversity in gene regulation, cytokine expression, and the effects of combinations of adjuvants were noted, with various antibody qualities showing a quantitative relationship to gene expression profiles across adjuvant compositions. Unfortunately, distinctions in Ig isotype and subclass biology in NHP, other animal models, and humans^60^ limit the direct value of these prior adjuvant comparison studies. Nonetheless, these studies consistently point to the ability of adjuvants to impact antibody features and effector functions with potential mechanistic relevance to vaccine-mediated protection from HIV-1 infection, providing a strong rationale for comparative evaluation of immune responses elicited by diverse adjuvants in humans.

The AVEG015 phase I trial was a rare study in which participants were immunized with a single gp120 antigen and a panel of adjuvants^61^. Previously, serum samples from two arms of this study, the alum and liposomal MPL adjuvanted arms, were characterized, and differences in the titer, durability, α4β7-integrin receptor inhibition-binding, and antibody-dependent cellular cytotoxicity (ADCC) activity were seen with adjuvant^62^. Here, the functional activity and biophysical features of the humoral response to immunization were characterized across the complete panel of adjuvants to assess the impact of adjuvant choice on response titer, durability, and non-neutralizing activity. Additionally, the balance of IgG and IgG3 to IgA subclasses elicited by immunization were compared to investigate the effect of adjuvant on this correlate of protection. Finally, linear regression analysis was employed to predict the functional activity of the responses from the biophysical features. Together the data and analyses presented here fill a gap in our current understanding of the ability of adjuvants to drive the humoral response human vaccination. Our findings support further exploration of adjuvants in improving the performance of HIV vaccines.

## Results

**Fig. 1:**
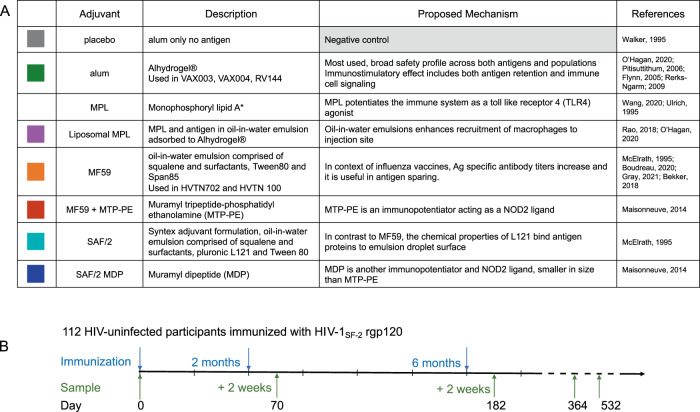
Vaccine regimen and adjuvants. **A** Table list the adjuvants used in the study. Colors in the left column will be used to define arms. *The MPL arm was excluded from analysis due to adverse effects. **B** Timeline depicts immunization and sample collection schedule.

The AVEG015 phase I trial enrolled 112 HIV-negative volunteers of both genders, aged 18-60, with low risk of HIV infection^61^. In addition to antigen, vaccines included one of seven different adjuvants: aluminum hydroxide (alum), monophosphoryl lipid A (MPL), liposome-encapsulated MPL with alum (liposomal MPL), MF59, MF59 plus muramyl tripeptide phosphatidylethanolamine (MTP-PE), syntex adjuvant formulation (SAF/2), and SAF/2 plus threonyl muramyl dipeptide (SAF/2 + MDP) (Fig. 1A). Volunteers were immunized three times at months 0, 2, and 6, and serum samples were collected at timepoint 0, 2 weeks after the second and third (final) immunizations, and at one and 1.5 years after the start of the trial (Fig. 1B). Functional activity and biophysical features of antibodies of the archived serum samples were measured using a novel and first in class approach to assess the functionality of antibody responses and the ability to engage FcR using Fc array. Previously, these samples were assayed for homologous neutralization activity^63^. For this study, serum samples from volunteers who received MPL adjuvanted vaccine are excluded from this analysis due to adverse reactions

### Antibody response magnitude and character

**Fig. 2:**
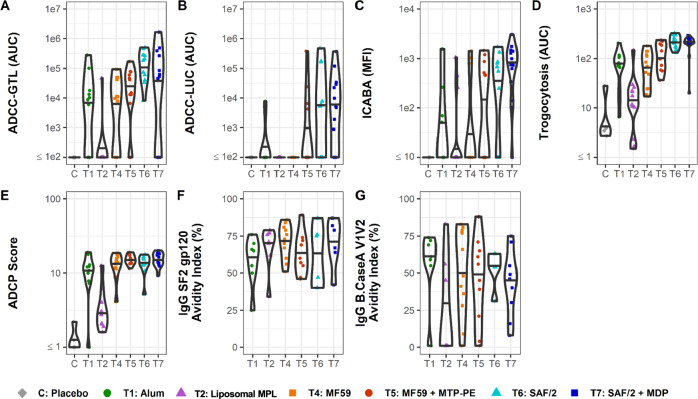
Antibody effector function and avidity 2 weeks post final vaccine dose. **A**–**D** Effector Functions. Antibody dependent cellular cytotoxicity-GranToxiLux (ADCC-GTL) area under the curve (AUC) (**A**), antibody dependent cellular cytotoxicity Luciferase (ADCC-LUC) area under the curve (AUC) (**B**), Infected Cell Antibody Binding Activity (ICABA) in mean/median fluorescence intensity (MFI) (**C**), Trogocytosis AUC (**D**), antibody-dependent cellular phagocytosis (ADCP) score (**E**), and antibody avidity to SF2 (**F**) and V1V2 from clade B Case A strain (**G**). Avidity (**F** and **G**) is shown only for participants with a positive vaccine-induced binding response (see Methods for details). Individual vaccine recipients are indicated by dots, and violins plots present group distributions, with median values indicated with a bar.

Non-neutralizing, Fc-mediated effector functions of the humoral response, particularly ADCC and ADCP, have correlated with decreased risk of infection in human HIV vaccine trials RV144 and HVTN 505, respectively^40,41^. To understand how adjuvants might affect the functional response to vaccination, effector functions of the vaccine-matched SF-2-specific antibodies were measured. Because antibodies mediate effector functions through FcγR interactions^64–66^, binding responses to FcγRs were also measured. At the peak of the humoral response, 2 weeks after the final immunization, vaccines adjuvanted with MF59 + MTP-PE, SAF/2, and SAF/2 + MDP increased the magnitude of antibody responses that mediate ADCC (both GranToxiLux (GTL) and Luciferase (LUC) based assays), infected cell binding (assessed by infected cell antibody-binding assay, ICABA), trogocytosis, and ADCP in comparison to alum (Fig. 2A–E). Compared to alum, MF59 drove similar extra-neutralizing activity across these measures. Antibodies raised by volunteers in the liposomal MPL arm conferred weaker extra-neutralizing activity compared to volunteers in the alum or other arms. In the RV144 trial, IgG avidity to the HIV envelope inversely correlated with infection risk^40,67^. Here, volunteers in all arms raised antibodies with comparable IgG avidity to SF-2 gp120 and CaseA2 V1V2, with the exception of volunteers in the liposomal MPL arm, whose antibodies had weaker median avidity to CaseA2 V1V2 loops compared to that of other arms (Fig. 2F and G). Together, data from this panel of assays reveals enhanced functional responses driven by the well-defined oil-in-water emulsion adjuvants, MF59 + MTP-PE, SAF/2, and SAF/2 + MDP, at peak immunogenicity as well as at later timepoints (Supplemental Figure 1).

**Fig. 3:**
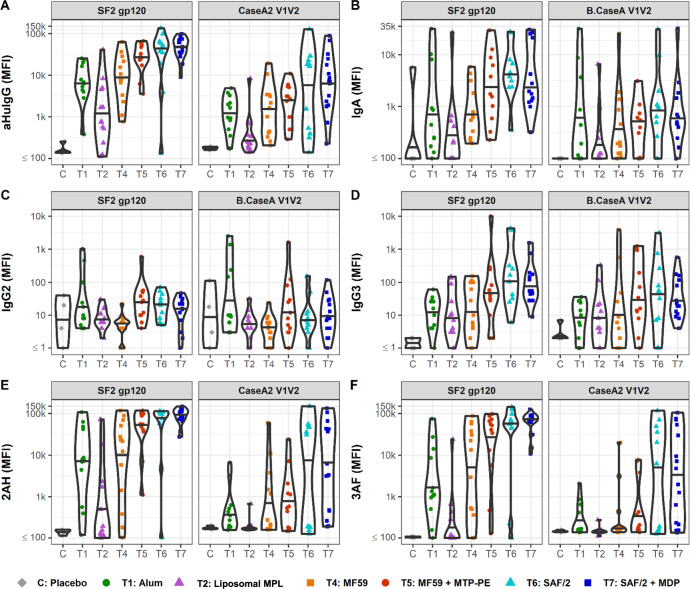
Antibody responses at 2 weeks past final vaccine. **A**–**F** Fc Array characterization of antibodies raised against SF2 and Case A V1V2. The isotype and subclass (**A**–**D**) and Fcγ receptor binding (**E**, **F**) profiles of HIV-specific antibodies are presented. 2AH indicates binding to FcγRIIAH, and 3AF indicates binding to FcγRIIIAF. Median fluorescence intensity (MFI) values for antibodies from individual vaccine recipients are indicated by dots, and violins plots present group distributions, with median values indicated with a bar.

The biophysical features of antibody responses to immunization were characterized by multiplexed assays^68,69^. The majority of volunteers raised SF-2-specific antibodies in response to immunization as compared to placebo (Fig. 3). SF-2-specific IgG and IgA responses peaked in all arms 2 weeks after the final immunization (Supplemental Figure 2). At this timepoint, volunteers in the MF59 + MTP-PE, SAF/2, and SAF/2 + MDP adjuvanted arms raised increased SF-2- and CaseA2 V1V2-specific IgG and IgG3 responses in comparison to alum (Fig. 3A and D, Table 1). These adjuvants also increased SF-2-specific IgA responses, while CaseA2 V1V2-specific IgA responses remained similar to alum (Fig. 3B). Volunteers who received liposomal MPL had weaker SF-2- and CaseA2 V1V2-specific IgG and IgA responses compared to alum. Compared to those in the alum arm, volunteers in the MF59 group had similar SF-2- and CaseA2 V1V2-specific responses across subclasses. Across adjuvants, median IgG2 SF2 and CaseA2 V1V2-specific responses were similar, with few volunteers raising high responses (Fig. 3C). Fcγ receptor binding was also assessed by Fc array (Fig. 3E and F). At the peak response, strong SF-2- and CaseA2 V1V2-specific FcγRIIa-binding responses were raised by SAF/2 and SAF/2 + MDP adjuvanted vaccines (Fig. 3E and F). MF59 + MTP-PE adjuvant also boosted SF-2-specific FcγRIIa and FcγRIIIa-binding responses compared to alum. These FcγR-binding responses parallel the increased ADCC and ADCP activity of the responses raised by volunteers in the MF59 + MTP-PE, SAF/2, and SAF/2 + MDP arms.

**Table 1:** Functional antibody response summary.

	Day 0	Day 182	Day 532
ADCC-GTL(AUC)			
C: Placebo	1.00 (1.00, 1.00)	1.00 (1.00, 1.00)	1.00 (1.00, 1.00)
T1: Alum	1.00 (1.00, 1.00)	8689.60 (1.00, 15595.38)	1.00 (1.00, 1.00)
T2: Liposomal MPL	1.00 (1.00, 1.00)	1.00 (1.00, 1.00)	1.00 (1.00, 1.00)
T4: MF59	1.00 (1.00, 1.00)	9638.29 (1.00, 41686.94)	1.00 (1.00, 1.00)
T5: MF59 + MTP-PE	1.00 (1.00, 1.00)	28055.91 (11376.78, 64227.95)	1.00 (1.00, 1026.75)
T6: SAF/2	1.00 (1.00, 1.00)	184077.20 (50194.54, 277732.64)	9155.15 (1706.60, 42571.92)
T7: SAF/2 + MDP	1.00 (1.00, 1.00)	49373.10 (1.00, 223094.62)	3507.78 (1.00, 8211.18)
ADCC-LUC (AUC)			
C: Placebo	1.00 (1.00, 1.00)	1.00 (1.00, 1.00)	1.00 (1.00, 1.00)
T1: Alum	1.00 (1.00, 1.00)	1.00 (1.00, 1.00)	1.00 (1.00, 1.00)
T2: Liposomal MPL	1.00 (1.00, 1.00)	1.00 (1.00, 1.00)	1.00 (1.00, 1.00)
T4: MF59	1.00 (1.00, 1.00)	1.00 (1.00, 1.00)	1.00 (1.00, 1.00)
T5: MF59 + MTP-PE	1.00 (1.00, 1.00)	1.00 (1.00, 4802.65)	1.00 (1.00, 1.00)
T6: SAF/2	1.00 (1.00, 1.00)	5432.50 (1.00, 86271.52)	1.00 (1.00, 29364.92)
T7: SAF/2 + MDP	1.00 (1.00, 1.00)	5026.30 (221.52, 27427.18)	1.00 (1.00, 6627.65)
ICABA (MFI)			
C: Placebo	0.00 (0.00, 0.00)	0.00 (0.00, 0.00)	0.00 (0.00, 100.25)
T1: Alum	0.00 (0.00, 0.00)	0.00 (0.00, 165.50)	0.00 (0.00, 217.75)
T2: Liposomal MPL	0.00 (0.00, 0.00)	0.00 (0.00, 64.25)	0.00 (0.00, 0.00)
T4: MF59	0.00 (0.00, 0.00)	0.00 (0.00, 0.00)	0.00 (0.00, 0.00)
T5: MF59 + MTP-PE	0.00 (0.00, 0.00)	229.50 (0.00, 604.50)	0.00 (0.00, 0.00)
T6: SAF/2	0.00 (0.00, 0.00)	672.00 (124.00, 831.00)	70.00 (0.00, 965.25)
T7: SAF/2 + MDP	0.00 (0.00, 0.00)	820.00 (482.50, 1409.00)	82.00 (0.00, 596.50)
ADCP Score			
C: Placebo	1.48 (1.06, 2.18)	1.00 (0.75, 1.49)	1.02 (0.80, 1.41)
T1: Alum	1.55 (1.10, 2.19)	11.45 (8.34, 12.74)	2.30 (1.48, 3.02)
T2: Liposomal MPL	1.70 (1.15, 2.10)	2.88 (2.06, 3.76)	1.70 (1.50, 2.10)
T4: MF59	1.45 (1.20, 2.25)	13.75 (11.60, 16.23)	5.03 (2.95, 6.44)
T5: MF59 + MTP-PE	1.33 (0.95, 2.22)	15.18 (13.61, 16.90)	8.80 (7.66, 12.45)
T6: SAF/2	1.40 (1.16, 2.05)	13.50 (12.70, 15.85)	11.65 (11.43, 12.38)
T7: SAF/2 + MDP	1.35 (1.00, 2.54)	15.22 (13.45, 17.96)	10.40 (8.85, 14.35)
Trogocytosis (AUC)			
C: Placebo	12.65 (9.03, 16.94)	3.70 (3.30, 9.88)	
T1: Alum	6.36 (2.29, 12.31)	78.64 (55.73, 109.43)	
T2: Liposomal MPL	6.14 (1.85, 12.47)	11.69 (10.26, 22.09)	
T4: MF59	5.58 (2.17, 12.17)	69.78 (24.48, 119.60)	
T5: MF59 + MTP-PE	7.44 (4.08, 11.76)	120.80 (62.02, 195.42)	
T6: SAF/2	11.52 (5.16, 21.83)	218.00 (173.55, 277.10)	
T7: SAF/2 + MDP	7.37 (3.24, 8.64)	219.20 (206.02, 244.00)	

Median and interquartile range (25th, 75th percentiles) of functional response values by group at days 0, 182, and 532 (pre-immunization, 2 weeks post final immunization, and 1 year post final immunization, respectively). Trogocytosis was not run on Day 532 due to sample availability. ADCC-GTL and ADCP responses were measured to SF-2 gp120; ADCC-LUC and ICABA responses were measured to an SF162 infectious molecular clone; and trogocytosis responses were measured to BaL gp120.

**Fig. 4:**
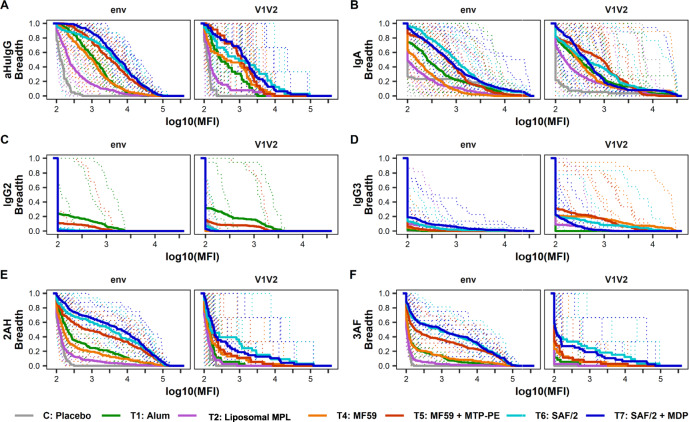
Antibody-binding breadth. Magnitude-breadth of binding antibody responses within subclass (**A–D**), Fcγ receptor binding (**E**, **F**), and Fab recognition (envelope or V1V2 loop) measured by Fc Array (**A**, **E**, and **F**) and BAMA (**B**, **C**, and **D**) assays at 2 week post the second vaccine. 2AH indicates binding to FcγRIIAH, and 3AF indicates binding to FcγRIIIAF. Breadth at each point is defined as the proportion of antigens within env and V1V2 antigen panels with log10(MFI) greater than or equal to each *x*-axis value. Antigen panels are listed in Supplemental Table 1, with the env panel including gp120 and gp140 antigens. Solid bold lines represent the median breadth for each arm, and the dotted lines are magnitude-breadth curves for each individual.

To characterize the antigen-binding fragment (Fab) response to vaccination across adjuvants, breadth scores were calculated for antibodies recognizing envelope and V1V2 antigens across subclasses and FcγRs (Fig. 4). Adjuvants drove distinct envelope recognition breadth. Compared to the alum arm, volunteers in the MF59 + MTP-PE, SAF/2, and SAF/2 + MDP arms elicited IgG and IgA responses with increased breadth of recognition of envelope antigens. In contrast, volunteers in the liposomal MPL arm elicited IgG and IgA responses with narrowed envelope binding breadth (Fig. 4A and B). The FcγRIIa and FcγRIIIa-binding antibodies have a similar pattern of envelope binding across adjuvants, with volunteers receiving SAF/2 and SAF/2 + MDP adjuvanted vaccines raising responses with the largest breadth (Fig. 4E and F). IgG and FcγR-binding responses raised by volunteers in the MF59 group had similar breadth to that of alum, but decreased the breadth of the envelope-specific IgA response compared to alum (Fig. 4A, B, E, and F). By many measures, the adjuvants did not broaden Fab recognition of V1V2 loops compared to alum with two important exceptions: SAF/2 and SAF/2 + MDP immunization increased the breadth of V1V2-specific antibodies that bound FcγRIIIa (Fig. 4F), and MF59 and MF59 + MTP-PE immunization increased the breadth of IgG3 antibodies that bound V1V2 antigens (Fig. 4D).

**Fig. 5:**
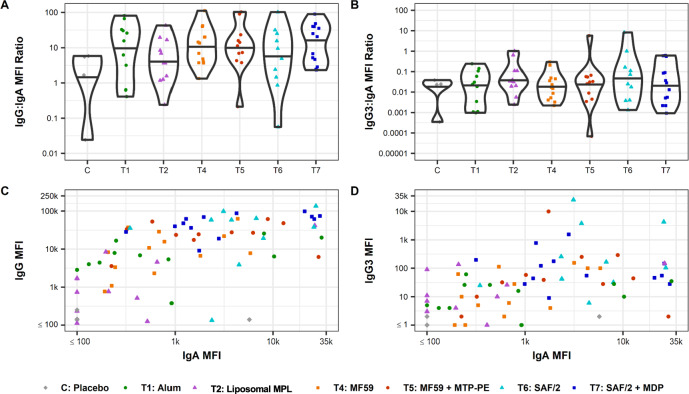
Relative differences in induction of desirable versus undesirable antibody types. Ratio of MFI determined by Fc Array of SF-2 gp120-specific antibodies raised 2 weeks past final vaccination for subclass IgG over IgA (**A**) and IgG3 over IgA (**B**). Individual vaccine recipients are indicated by dots, and violin plots present group distributions, with median values indicated with a bar (**A**, **B**). Scatterplots of MFI values determined by Fc array indicate IgA vs IgG (**C**) or IgA vs IgG3 (**D**) elicited against SF-2 at 2 weeks past final vaccination. Dots represent individual vaccine recipients; color and shape indicate study arm.

Because analysis of the RV144 and HVTN 505 trials suggests that the profile of robust envelope specific IgG and IgG3 responses in the absence of certain envelope specific IgA responses correlates inversely with infection risk^41,43–46^, the ratios of IgG and IgG3 to IgA were calculated. Because of the small number of subjects in each arm and the variability of responses within arms, we chose to compare these ratios qualitatively. On this basis, the ratios of these measures did not vary with adjuvant (Fig. 5A and B). To further explore the character of the IgG and IgG3 to IgA responses, these measures were plotted against each other (Fig. 5C and D). Across participants, changes to the balance of IgG to IgA or IgG3 to IgA did not appear to be strongly modified by adjuvant. The responses seen here suggest that adjuvants may increase the titer of the response across subclasses without consistently affecting the proportion of IgG to IgA or IgG3 to IgA.

### Antibody functions over time

One critical metric of successful HIV vaccination will be the durability of the response and protection. Studies in NHP suggest that adjuvant may affect the durability of the HIV-specific antibody responses to immunization^59^. Both biophysical and functional assays were performed on samples collected up to a year after the final vaccination. Compared to alum, MF59 + MTP-PE, SAF/2, and SAF/2 + MDP adjuvanted immunization led to more durable SF-2-specific IgG and SF-2-specific FcγRIIa- and FcγRIIIa-binding antibodies up to a year past the final immunization (Supplemental Figure 2A, E, and F, Table 2). SF-2-specific IgG3 responses remained elevated 6 months and a year after final vaccination in the SAF/2 and SAF/2 + MDP arms compared to alum (Supplemental Figure 2D, Table 2). In addition, the SF-2 specific IgA responses in these two arms was elevated a year after immunization (Supplemental Figure 2B, Table 2). The durability of the functional response will also be important for effective HIV vaccination. In all arms, ADCC, by both GTL and LUC assay, and ICABA activity declined after the peak humoral response, 2 weeks after the final vaccination (Supplemental Figure 1A–C, Table 1). Interestingly, immunization with MF59, MF59 + MTP-PE, SAF/2, and SAF/2 + MDP led to increased ADCP activity up to a year past the final vaccination (Supplemental Figure 1E, Table 1).

**Table 2:** SF-2 gp120 Binding antibody response summary.

	Day 0	Day 182	Day 364	Day 532
IgG				
C: Placebo	116 (114, 118)	141 (139, 170)	124 (111, 137)	106 (95, 119)
T1: Alum	110 (103, 115)	6618 (4302, 17518)	1126 (319, 2459)	573 (262, 1770)
T2: Liposomal MPL	106 (100, 112)	766 (514, 4551)	404 (192, 988)	247 (162, 490)
T4: MF59	100 (98, 114)	8274 (3319, 21896)	2369 (1417, 3031)	1855 (1045, 2657)
T5: MF59 + MTP-PE	108 (104, 120)	27247 (22087, 49264)	5770 (2732, 9170)	4575 (1854, 7715)
T6: SAF/2	93 (92, 104)	52246 (27353, 61270)	11504 (9118, 20394)	9286 (6851, 13089)
T7: SAF/2 + MDP	117 (102, 122)	54494 (35462, 69781)	9532 (6708, 17217)	7528 (5383, 12553)
IgA				
C: Placebo	50 (43, 1455)	62 (39, 1496)	60 (39, 1337)	54 (39, 1423)
T1: Alum	78 (45, 494)	440 (206, 4510)	357 (82, 1504)	258 (77, 698)
T2: Liposomal MPL	92 (72, 324)	197 (92, 467)	94 (86, 179)	101 (79, 524)
T4: MF59	74 (63, 128)	616 (246, 1809)	190 (136, 399)	150 (104, 274)
T5: MF59 + MTP-PE	179 (85, 568)	1737 (803, 7551)	704 (289, 2462)	839 (306, 2986)
T6: SAF/2	118 (30, 176)	4133 (2557, 7674)	574 (287, 612)	468 (229, 906)
T7: SAF/2 + MDP	140 (109, 270)	1958 (1292, 21030)	609 (432, 1858)	496 (292, 1658)
IgG2				
C: Placebo	6 (3, 16)	12 (3, 25)	5 (3, 28)	6 (3, 24)
T1: Alum	6 (6, 12)	10 (6, 62)	5 (4, 9)	8 (3, 16)
T2: Liposomal MPL	5 (4, 6)	6 (6, 14)	6 (4, 8)	7 (4, 11)
T4: MF59	4 (1, 6)	6 (4, 6)	4 (2, 5)	4 (4, 4)
T5: MF59 + MTP-PE	5 (2, 33)	26 (10, 51)	12 (4, 20)	9 (4, 16)
T6: SAF/2	5 (2, 11)	22 (10, 38)	12 (7, 32)	10 (6, 23)
T7: SAF/2 + MDP	6 (4, 12)	19 (8, 25)	10 (8, 15)	9 (4, 12)
IgG3				
C: Placebo	2 (1, 2)	2 (1, 2)	2 (1, 2)	2 (1, 3)
T1: Alum	1 (1, 3)	16 (4, 27)	2 (1, 4)	2 (1, 3)
T2: Liposomal MPL	2 (2, 4)	8 (3, 42)	4 (1, 8)	4 (1, 10)
T4: MF59	1 (1, 1)	10 (4, 100)	2 (1, 3)	2 (1, 3)
T5: MF59 + MTP-PE	1 (1, 2)	42 (24, 124)	5 (1, 13)	2 (1, 8)
T6: SAF/2	1 (1, 2)	166 (37, 2056)	26 (8, 66)	14 (7, 45)
T7: SAF/2 + MDP	1 (1, 2)	88 (44, 191)	10 (5, 53)	8 (3, 39)
2AH				
C: Placebo	113 (111, 114)	147 (131, 158)	131 (123, 140)	122 (107, 134)
T1: Alum	122 (110, 136)	7726 (3466, 43445)	182 (141, 248)	150 (117, 224)
T2: Liposomal MPL	141 (121, 158)	153 (117, 1707)	123 (113, 146)	126 (116, 152)
T4: MF59	122 (112, 133)	21434 (1420, 50664)	250 (170, 498)	279 (152, 470)
T5: MF59 + MTP-PE	121 (101, 164)	54926 (39344, 98460)	3587 (298, 6840)	2638 (272, 9102)
T6: SAF/2	109 (106, 110)	97338 (66302, 108843)	12354 (8101, 35026)	13982 (8377, 20958)
T7: SAF/2 + MDP	126 (117, 143)	106868 (72747, 121201)	8345 (3286, 28480)	7881 (1844, 34080)
3AF				
C: Placebo	115 (106, 124)	106 (103, 108)	109 (104, 112)	114 (102, 128)
T1: Alum	104 (98, 111)	1234 (841, 9957)	105 (98, 116)	116 (104, 126)
T2: Liposomal MPL	117 (110, 122)	108 (100, 280)	98 (90, 109)	112 (97, 114)
T4: MF59	107 (97, 116)	13195 (499, 37570)	123 (102, 178)	118 (110, 164)
T5: MF59 + MTP-PE	107 (100, 115)	46103 (11138, 67220)	455 (114, 1452)	483 (137, 1089)
T6: SAF/2	107 (105, 107)	56160 (44682, 83931)	3150 (825, 13164)	1851 (1478, 10220)
T7: SAF/2 + MDP	108 (94, 118)	71527 (62141, 89254)	1511 (488, 8908)	2654 (560, 12915)

Median and interquartile range (25th, 75th percentiles) of binding response values by group at days 0, 182, 364, and 532 (pre-immunization, 2 weeks post final immunization, 7 months post final immunization, and 1 year post final immunization, respectively). IgG, 2AH (FcγRIIAH), and 3AF (FcγRIIIAF) responses were measured by Fc array, and IgA, IgG2, and IgG3 responses were measured by BAMA.

### Modeling antibody functions based on biophysical profiles

**Fig. 6:**
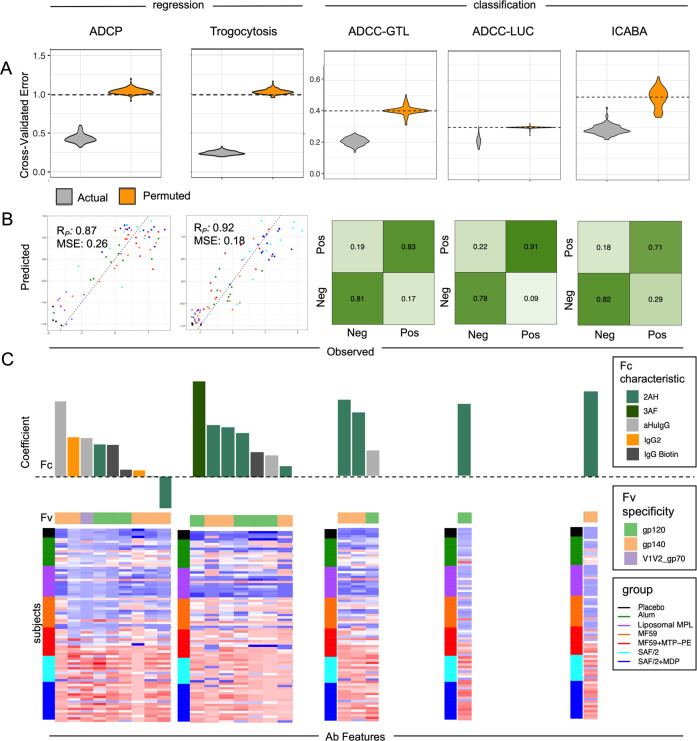
Predictions of antibody functions based on biophysical features. **A** Mean cross-validated error observed in predicting diverse antibody functions as continuous variables (regression, left) or by class (high/low activity classification, right). Performance of repeated modeling of actual study data is shown in gray violin plots. Predictive repeated modeling of permuted activity magnitudes (ADCP, trogocytosis) or class labels (ADCC-GTL, ADCC-LUC, ICABA) is shown in orange violin plots, and performance does not overlap with modeling of actual data, *p*-values < 0.01. The mean error expected at random is indicated by dotted lines. **B** Performance of the final model for different functional outcomes as defined by Pearson correlation coefficient (R_P_) and mean squared error (MSE) for (ADCP, trogocytosis) or by confusion matrix (ADCC ADCC-GTL, ADCC-LUC, ICABA) between predicted and observed activity. **C** Features contributing to models of antibody function. In the bar chart at top, features are arranged by the magnitude and direction of their coefficients (top), with Fc characteristic indicated in color, and the Fv specificity of each feature shown in the color bar below. The dotted line indicates a coefficient of zero. In the heatmap at the bottom, responses observed across vaccine recipients in each study group are shown for each feature (column) contributing the model. Subjects (rows) are grouped by adjuvant. High responses are shown in red, and low responses in blue. 2AH indicates binding to FcγRIIAH, and 3AF indicates binding to FcγRIIIAF.

In the past decades, supervised learning techniques were developed and applied to biological and biomedical research with increasing prevalence. Compared to traditional univariate correlation analysis between variables and outcomes, multivariate modeling approaches have demonstrated their capability in prediction with a combination of multiple variables and validation in independent cohorts^41,54^. With previous success in identifying factors that contributed to functional activity, we sought to use multivariate modeling approaches to predict antibody effector functions among the vaccinated subjects from the biophysical measures characterized by the Fc Array assay^44,54,70^ at the peak humoral response, 2 weeks after the final immunization. Based on the natural distribution of the various functional assay readouts, a linear regression model was chosen to predict the continuous measurements of ADCP and trogocytosis, while a logistic regression classifier was chosen to predict the binary outcome (low or high activity) of ADCC-GTL, ADCC-LUC, and ICABA (details in Methods). Repeated modeling enabled the comparison of cross-validated error between models trained on actual and permuted functional assay data, establishing confidence in the robustness and generalizability of models for each antibody activity (Fig. 6A).

To evaluate the biophysical antibody features supporting predictions of each functional response, final models were trained (Fig. 6B). For regression models, Pearson’s correlation coefficient and mean squared error (MSE) were computed between the observed and predicted responses to evaluate model quality (ADCP: Rp = 0.87, MSE = 0.26; trogocytosis: Rp = 0.92, MSE = 0.18), whereas confusion matrices and accuracies are reported for logistic regression models (ADCC-GTL–accuracy = 0.83, f1 = 0.80; ADCC-Luc–accuracy = 0.83, f1 = 0.86; ICABA–accuracy = 0.76, f1 = 0.78).

As the features were standardized before modeling, the relative magnitude of the feature coefficients could be considered as the surrogate of the rank of importance within each model (Fig. 6C). Not surprisingly, responses with high titers of antibodies that bound FcγRIIa and gp120 or gp140 were the most frequently high-ranked predictors of different types of functional responses, followed by responses with high titers of gp120 or gp140-specific IgG and antibodies that bound FcγRIIIa.

### WGNCA to study the association between antibody features in the Fc array dataset

One limitation of the modeling approach applied is that it may eliminate variables that are highly correlated with selected features in order to optimize model simplicity and to avoid overfitting. Thus, a network analysis was conducted to identify both the biophysical features that are correlated to the selected predictors and the biophysical features’ association with other variables in the profile.

**Fig. 7:**
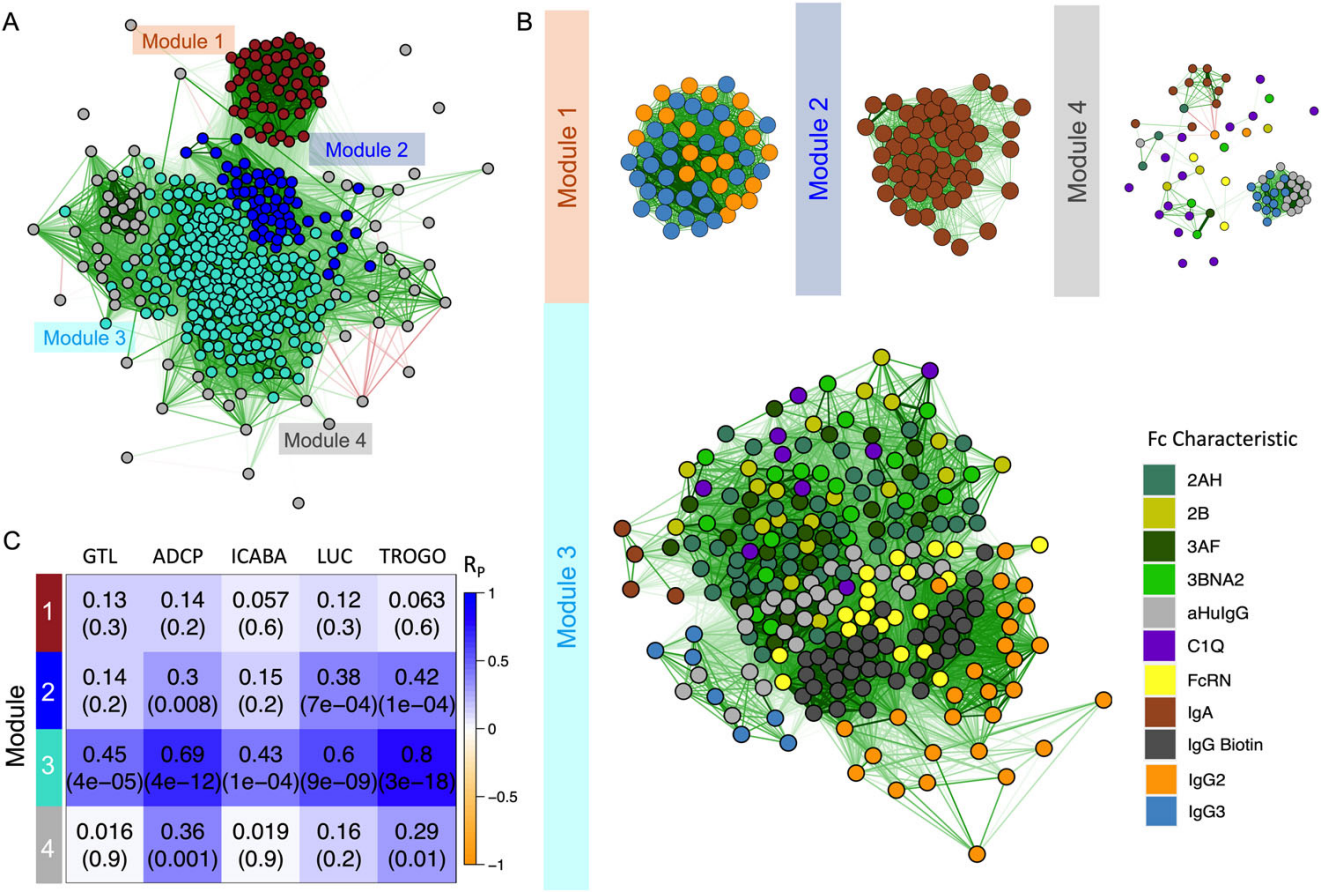
Multiple antibody functions are associated with a module of specific antibody features. **A**, **B** Feature correlation networks. Edges (green lines for positive correlation, red lines for negative correlation) between nodes depict relationships with Pearson correlation coefficients (*R*_P_) < 0.3. **A** A weighted correlation network of antibody response feature modules was identified by hierarchical clustering and dynamic tree cutting. Features are colored by module and graphed according to degree and direction of correlation (R_P_). **B** Correlation network for features found within each module. Nodes (features) are colored by Fc characteristic. **C** Heatmap depicting correlative relationships between antibody functions (columns) and feature module eigenvalues (rows). Pearson correlation coefficients (R_P_) and *p*-values are shown in inset. 2AH indicates binding to FcγRIIAH, 2B indicates binding to FcγRIIB, 3AF indicates binding to FcγRIIIAF, and 3BNA2 indicates binding to FcγRIIIBNA2.

Designed to characterize the pair-wise correlation pattern of gene expression in high-dimensional transcriptomic datasets, weighted correlation network analysis (WGCNA) identified highly correlated antibody response features as modules (Fig. 7A and B). Among these, module 3 was well correlated with each antibody function assessed in this study (Fig. 7C), suggesting that it is composed of features of the immune response that drive polyfunctionality. Whereas module 1 was enriched with most IgG3 and IgG2 features in the biophysical measurements profile, module 2 was enriched with IgA responses, and module 3 contained FcγR-binding, C1Q, and other IgG features in a compact cluster (Fig. 7B).

## Discussion

Correlates analysis from HIV vaccine efficacy trials to date have highlighted both the characteristics and the Fc effector function of the humoral response as relevant to infection risk^40,41,71^. There have been limited opportunities to systematically assess how adjuvants influence the humoral response as most trials in both humans and NHP use only one or two adjuvants. The HVTN 702 trial used a vaccination regimen that included type a C ZM96 strain of gp120 and MF59 adjuvant with hopes of improving upon the performance of RV144, which had a vaccine regimen that included a different protein vaccine sequence and adjuvant, subtype CRF01_AE 92TH023 strain of gp120 and alum^72^. The disappointing lack of efficacy for HVTN 702^72^ underscores the need to connect each component of the immunization strategy, such as the choice of adjuvant, to the quality of the immune response. Our current study of HIV-specific antibody responses across six diverse adjuvants with the same immunogen reveals how antibody quality as well as magnitude is altered.

These six adjuvants interact with both antigen and the immune system in different ways. An important aspect of the mechanism of these adjuvants is signaling through innate immune cell pathogen recognition receptors (PRR)^73^. PRRs respond to both damage associated molecular patterns (DAMPs), molecules released during host cell damage or death, and pathogen associated molecular patterns (PAMPs), molecules derived from viruses, bacteria, and fungi^73,74^. Alum and the oil-in-water emulsion adjuvants, MF59 and SAF/2, enhance immune signaling through DAMPs, by causing limited tissue damage at the site of the reaction^75^. DAMP signaling recruits APCs to the site of injection, increasing uptake of antigen and trafficking to the draining lymph node^76,77^. While this signaling enhances the immune response to vaccination, DAMP signaling may fail to fully trigger responses that target intracellular pathogens^74,78^. The bacterial derived adjuvants, MPL, MTP-PE, and MDP, signal the immune system through PAMPs mainly via two receptors: MPL is interacts with TLR4^79,80^, and MTP-PE and MDP bind both TLR and NOD-like receptors^81^. The cytokines released upon injection of PAMP molecules can favor Th1 responses as compared to Th2 responses often seen with alum injection^82^. Combining adjuvants to include a PAMP molecule, oil-in-water emulsion, and/or alum, has been a successful strategy for diversifying immune signaling with vaccination while maintaining the necessary safety profile^59,73,83,84^. Another advantage of combinatorial adjuvant systems may be that alum and/or the oil-in-water emulsions help retain soluble antigen and immunopotentiating molecules at the site of injection and, in the case of MF59, at the lymph node^85,86^.

In this analysis, we see the most consistently enhanced effector functionality, antibody titer, and durability of the humoral response in the combinatorial SAF/2 + MDP-PE adjuvanted arm. MF59 + MDP and SAF/2 alone drove similar humoral responses to SAF/2 + MDP-PE by many measures including ADCP activity, CaseA2 V1V2-specifc IgG3 responses, and the magnitude and durability of the SF-2-specific IgG and FcγRIIa-binding responses. None of the adjuvants consistently affected the balance of subclass raised in response to immunization; rather volunteers with increased titers of SF-2-specific IgG or IgG3 also had increased titers of IgA.

Differences in the biophysical and functional characteristics of responses seen in studies of these adjuvants in either animal HIV vaccination models or in human vaccination with other antigens point to the need to carefully assess the effect of particular adjuvant and antigen formulations in humans. In the AVEG015 trial, MF59-adjuvanted immunization raised responses that were qualitatively similar in both biophysical and functional character to that of alum. Variable functional responses have been seen with MF59 in NHP HIV vaccination models. Vaccari et al. reported that MF59-adjuvanted vaccine drove enhanced Fc effector functionality compared to alum^56^. More consistent with the effect seen here, Francica et al. saw that use of MF59 adjuvant did not increase ADCC or antibody-dependent complement deposition (ADCD) and only slightly increased ADCP compared to alum^59^. In people given H5N1 influenza vaccine adjuvanted with alum or MF59, MF59 boosted titers of IgG1, IgG3 and FcγRIIa-binding H5-specific responses^87^. Here, liposomal MPL immunization raised weaker functional or biophysical responses compared to alum. Rao et al. also measured weaker ADCC activity in the liposomal MPL arm than in the alum arm using these samples^62^. However, this result is somewhat surprising as MPL adjuvanted HIV vaccination in model animals leads to increased humoral and functional responses^55,88^, and further emphasizes the importance of human trials and careful characterization of the humoral response to vaccination with different combinations of antigens and adjuvants. Further insights into the combinatorial effect of antigen and adjuvant will be gained from the results of HVTN 705 and 706, phase 2b trials of regimens including Ad26-delivered mosaic antigens as a prime and clade C gp140 (HVTN 705) or clade C and mosaic gp140 (HVTN 706) both adjuvanted with alum as boost^89^. These results will be of particular interest in the context of this study as initial testing of the regimen indicated that responses to these vaccines are weakly neutralizing and rely on functional antibodies and T cell responses for protection^90^.

Generalized linear regression models identified biophysical features that predicted functional outcome. Functional responses to vaccination, including ADCC, ADCP, ICABA, and trogocytosis, were predicted by envelope-specific FcγRIIa-binding antibodies raised at the peak of the humoral response. Modeling to predict functional outcomes was also partially driven by envelope-specific IgG and FcγRIIIa-binding antibody responses. These functionally predictive response features were strongly connected through network analysis to other HIV-specific IgG response features. Many of these features, including V1V2-specific IgG, FcγR binding, and complement activation, for which C1Q binding serves as a surrogate, have been identified as inversely correlated with infection risk in analysis of RV144^40,43,71^. In this analysis, envelope-specific IgG responses correlated well with ADCP activity, which has also correlated with protection from infection in a number of NHP vaccine studies^51,53–55^. On the other hand, correlation of IgG2 responses with phagocytosis was unexpected given the generally lower activity of this subclass, although increased HIV-specific IgG2 was associated with delayed HIV disease progression in individuals who also expressed an FcγRIIa allele capable of interacting with IgG2^91,92^. Taken together, while inter-subject variability was high across groups, and the study was underpowered to develop adjuvant-specific humoral response networks and evaluate their similarities or differences, these results confirm that choice of adjuvant influences the titer, breadth, durability, and functionality of the humoral response to immunization. It will be of interest to compare the humoral response networks observed in other human HIV vaccine studies to that observed here, as well as to define adjuvant-specific network signatures.

Creating a protective HIV vaccine presents a significant challenge. Natural infection is not protective, and broadly neutralizing antibodies (bnAbs) are raised slowly in only a subset of individuals^29^. Likewise, it has thus far been impossible to raise durable bnAbs by vaccination^93^. Partial protection seen in HIV trials has been linked to the Fc effector function and biophysical features of the humoral response. Unfortunately, the partial protection raised in RV144 waned over time^94,95^, and was not recapitulated in HVTN 702 and 705 follow up studies that aimed to reproduce and improve upon this level of VE^72,95^. Similar analysis of antibody responses in these trials, paired with case-control studies will be needed in order to help define the prospects of functional antibody-based vaccine concepts. It is clear that optimization of each aspect of the vaccination regimen may be needed for success. Beyond well-known effects on titer and durability, this study shows that adjuvants can tailor the biophysical features and effector functions of the immune response. These results represent a rare opportunity to observe the effect of many adjuvants in human volunteers, and this finding bodes well for ongoing work that employs new adjuvants or new applications of current adjuvants^96,97^.

## Methods

### Samples

Volunteers in the experimental arms were given three doses of 50 mcg of HIV-1_SF2_ rgp120 (biocine) combined with one of the seven adjuvants at months 0, 2, and 6 (Days 0, 56, and 168)^61^ (Clinical Trials.gov NCT00001042). In the placebo arm, volunteers were given alum only. Volunteers were followed for 1 year after the last injection, and blood samples were collected pre-injection (day 0), 2 weeks after the second and third (final) immunization (days 70 and 182), and at longer timepoints a year after the first immunization and a year after the final immunization (Days 365 and 532). Serum samples from volunteers who received MPL adjuvanted vaccine are excluded from this analysis due to adverse reactions. Ethical approval for the clinical study was obtained by each clinical site, including Saint Louis University, University of Rochester, University of Washington, and Vanderbilt University. Participants provided written informed consent. Ethical approval for the analyses included here was provided by Duke University.

### Biophysical antibody profiles

Two multiplexed assays were used to characterize the biophysical features of HIV-specific antibodies. IgG titer and the ability to interact with human FcγR’s (including FcγRIIAH, FcγRIIB, FcγRIIIAF, FcγRIIIBNA2), FcαR, and FcRN, and C1q were measured by Fc Array^68^. IgG2, IgG3, and IgA titers were determined by binding antibody multiplexed assay (BAMA)^69,98^. IgG avidity was measured by a BAMA Avidity Index assay^99^. For Fc array, BAMA, and BAMA Avidity Index assays, a diverse panel of HIV antigens (Supplemental Table 1) was covalently coupled to fluorescently coded beads (Luminex Corp.). Pooled, antigen-coupled beads were then incubated with dilute serum samples. Following incubation, samples were washed, and antigen bound antibodies were detected with phycoethrin (PE)-labeled anti-human subclass-specific secondary antibodies or tetramerized protein, in the case of Fc receptors and C1Q^54,68^. The fluorescent code of the bead and the PE fluorescence were simultaneously detected by FlexMap three-dimensional (3D) array reader. Median fluorescence intensities were calculated for each bead type. For Fc Array, pooled intravenous immunoglobulin from HIV positive patients (HIVIG) and CH31 IgA (an HIV-specific mAb) titration curves were used as controls to define the define bead antigenicity profiles. For BAMA, HIVIG titration curves were run to control for antigen-coupled bead performance. BAMA binding responses were considered positive if post-vaccination MFI was greater than 100, greater than the antigen-specific cutoff (95^th^ percentile of baseline responses by isotype and antigen), and greater than 3-fold over subject-specific baseline response. BAMA avidity index is the proportion of binding magnitude retained in the presence of pH 3.0 sodium citrate buffer (AI = 100 × FI-bkgd (CIT)/FI-bkgd (PBS)) and is reported only for positive vaccine-induce responses within the linear range of the assay (100 < MFI < 23,000).

### Antibody-dependent cellular phagocytosis

Quantification of ADCP was performed by covalently binding SF-2 gp120 envelope glycoprotein to neutravidin fluorescent beads (ThermoFisher) and forming immune complexes by incubating in the presence of 1:50 dilution of serum^100–103^. Immune complexes were then incubated in the presence of monocyte THP-1 cells (TCC^®^ TIB-202^™^), and the fluorescence of the cells was detected using flow cytometry, calculating an ADCP score by multiplying the mean fluorescent intensity (MFI) and frequency of phagocytosis positive cells and then dividing by the MFI and frequency of the bead positive cells in an antibody negative control well. To assess sensitivity, two independent experiments were conducted and analyzed independently with similar results.

### Antibody-dependent cellular cytotoxicity

The ADCC-GranToxiLux (GTL) procedure was used to detect ADCC activity^104^. Recombinant HIV_SF-2_ gp120 was used to coat CEM.NKR_CCR5_ as target cells and PBMC obtained by leukapheresis from a healthy HIV-seronegative individual (Fc-gamma-Receptor IIIa 158 V/F phenotype) were used as source of effector cells. Effector and target cells (30:1 ratio) were plated in opaque 96-well plates and co-cultured with plasma dilutions of 1:50, 1:250, 1:1250, 1: 6250, 1:31,250 and 1: 156,250. The positivity of the assay was defined as ≥8% Granzyme B activity of the target cells after background subtraction.

For the ADCC-luciferase assay, CEM.NKR_CCR5_ cells were infected with HIV-1 SF162 Infectious Molecular Clone (GenBank accession number EU123924)^105,106^. Briefly, effector cells were obtained as for the GTL assay. The cells were thawed the day before the assay and rested overnight in RPMI 1640 medium supplemented with antibiotics and 10% fetal bovine plasma (R10), recombinant human IL-15 at a concentration of 10 ng/ml. Each plasma sample was assayed at six dilutions as indicated above, starting at a dilution of 1:50, in duplicate. Co-cultures were incubated for 6 h at 37 °C in 5% CO_2_. The assay readout is luminescence intensity (measured in relative light units, RLUs) generated by surviving target cells that have not been lysed by the effector population in the presence of ADCC-mediating plasma Abs. For the ADCC-luciferase assay, positive response to SF162 Luciferase is defined as having a net percent specific killing ≥10% at any of the first two dilutions. Previous work indicates that this threshold reduces the false positive rate to <2%^106^.

For both assays, the mAb palivizumab (Synagis), which mediates ADCC^107^ but is specific for respiratory syncytial virus, and a cocktail of HIV-1 mAbs (HIV-1 mAb mix) demonstrated to mediate ADCC [A32^108^, 2G12^109^, CH44^110^, and 7B2^111^] were used as negative and positive controls, respectively.

### Infected Cell Antibody-Binding Assay (ICABA)

Serum antibody binding to infected cells expressing HIV-1 Env was measured using indirect surface staining^108^. First, CEM.NKR_CCR5_ were infected with HIV-1 SF162 Infectious Molecular Clone (GenBank accession number EU123924) as described for the ADCC-Luciferase assay;^105,106^ these cells were subsequently incubated with vaccinees’ serum samples diluted 1:100. Cells were stained with a live/dead stain, then permeabilized and stained to detect human antibodies and HIV Env. Positive cells were defined as those with binding to ≥4% p24+ cells after subtracting the binding to mock-infected cells and binding of the baseline samples.

### Rapid Fluorometric Antibody-Dependent Cellular Trogocytosis (RFADCT) assay

Antibody-dependent trogocytosis was evaluated using our modification^112^ of the original Rapid Fluorometric Antibody-Dependent Cellular Cytotoxicity assay (RFADCC) originally described by Gomez-Roman and collaborators^113^. Our modification of the RFADCC assay uses the EGFP-CEM-Nkr^114^ target cell line engineered to co-express a Snap-tagged CCR5 on the cell surface (EGFP-CEM-Nkr-CCR5-SNAP). Briefly, EGFP-CEM-Nkr-CCR5-SNAP target cells were sensitized with gp120 of the HIV-1_BaL_ isolate as described^112^ and cultured for 2 h at 37 °C in the presence of peripheral blood mononuclear cells (PBMCs) from healthy donors and serum dilutions from vaccinated volunteers. RFADCT was quantified for replicate cultures by flow cytometry as described^112^. It should be noted that it is now known that the RFADCC assay largely detects antibody-mediated trogocytosis of immune complexes from sensitized target cells onto the surfaces of monocyte effector cells with cytotoxicity being a minor component of the observed effect^115–117^. For this reason, we now refer to our modified assay as the RFADCT assay.

### Modeling antibody functions

Biophysical measurements were filtered based on the number of missing values and positive responses and features were eliminated if >30% of values were missing or if <50% of subjects in the SAF-2-based regimens, which appeared to have the greatest overall immunogenicity, showed a response. For the remaining features, missing values were imputed by K-nearest-neighbors (KNN) with a selection *k* = 5 using the R package “bnstruct”^118^. Biophysical features were log_10_ transformed, centered and scaled before modeling.

Generalized linear and logistic regression models were employed to predict functional measurements with biophysical features. L1-norm penalization (LASSO) was applied to select features and improve prediction accuracy by minimizing sum of squares while constraining feature coefficients^119^. In order to predict ADCP and trogocytosis scores, a linear regression was used. To minimize the potential of over or underfitting, the parameter, λ, was tuned with 5-fold cross validation over 100 times repeated modeling, a final model was trained on all data with λ set at the largest value such that median squared error (MSE) was within 1 standard error of the minimum (λ.1se), and Pearson’s Correlation Coefficients were calculated between predicted and observed activity. Given their more bimodal activity distributions, a weighted logistic regression with L1 penalty was chosen to predict GTL, LUC, and ICABA, where F1 (the harmonic mean of precision and recall) was employed as the evaluation metric for model selection. To further evaluate model robustness and reliability, permutation tests were employed, and the error observed following repeated modeling when functional activities were randomized across subjects was reported. The R package “glmnet” was employed for the analysis^120^.

### Weighted correlation network analysis

Using the “WGCNA” package, a weighted correlation network was constructed with biophysical features from the Fc array and BAMA assays^121^. Variables and subjects with either too many missing entries or zero variance were removed with the function “goodSamplesGenes” at verbose = 3. A soft threshold power was set at 9, and a minimum module size of 25 was selected to cluster the network and identify modules from the biophysical features with dynamic tree cutting algorithm. The summary profile for each identified module was correlated with each functional measurement. The network visualization of weighted correlation was graphed with R package “qgraph”^122^; the heatmap of the Pearson’s correlation coefficient between each biophysical measurement and functional measurement was generated with R package “heatmap3”^123^.

### Reporting summary

Further information on research design is available in the Nature Research Reporting Summary linked to this article.

## Supplementary information

Supplemental Information

REPORTING SUMMARY

## Data Availability

Data is available from study authors upon reasonable request.
