# Supplementary material for: Impact of adjuvants on the biophysical and functional characteristics of HIV vaccine-elicited antibodies in humans
Source: NPJ Vaccines. 2022 Aug 4;7:90. doi: 10.1038/s41541-022-00514-9 (PMC9352797; doi:10.1038/s41541-022-00514-9)
Supplement: Supplementary file 1 — Supplemental Information [file 41541_2022_514_MOESM1_ESM.pdf]

Supplemental Information

Impact of adjuvants on the biophysical and functional characteristics of HIV vaccine-elicited antibodies in humans

Shiwei Xu^[1], Margaret C. Carpenter^[2], Rachel L. Spreng[3], Scott D. Neidich[3], Sharanya Sarkar[2], DeAnna Tenney[3], Derrick Goodman[3], Sheetal Sawant[3], Shalini Jha[3], Brooke Dunn[3], M. Juliana McElrath[4], Valerie Bekker[3], Sarah V. Mudrak[3], Robin Flinko[5], George K. Lewis[5], Guido Ferrari[3], Georgia D. Tomaras[3], Xiaoying Shen[3*], Margaret E. Ackerman[1,2]*

[1]Quantitative Biomedical Science Program, Dartmouth College, Hanover, NH USA
[2]Thayer School of Engineering, Dartmouth College, Hanover, NH, USA
[3]Department of Surgery, Duke University School of Medicine, Durham, North Carolina Duke Human Vaccine Institute, Duke University Medical Center, Durham, NC, USA
[4]Vaccine and Infectious Disease Division, Fred Hutchinson Cancer Research Center, Seattle, WA, 98109, USA; Departments of Laboratory Medicine and Medicine, University of Washington, Seattle, WA, USA.
[5]Division of Vaccine Research, The Institute of Human Virology, University of Maryland School of Medicine, Baltimore, MD, USA

^ Co-first authors

*Corresponding authors

Xiaoying Shen
915 S. LaSalle St. Durham, NC 27710
sxshen@duke.edu
919 684 9986

Margaret E. Ackerman
14 Engineering Dr,
Hanover, NH 03755
margaret.e.ackerman@dartmouth.edu
603 646 9922

**Supplemental Figures**	
Supplemental Figure 1	Longitudinal profile of SF2-specific antibody functions
Supplemental Figure 2	Longitudinal profile of SF-2-specific antibodies
**Supplemental Tables**	
Supplemental Table 1	Antigens used in BAMA/BAMA-AI and Fc Array Assays

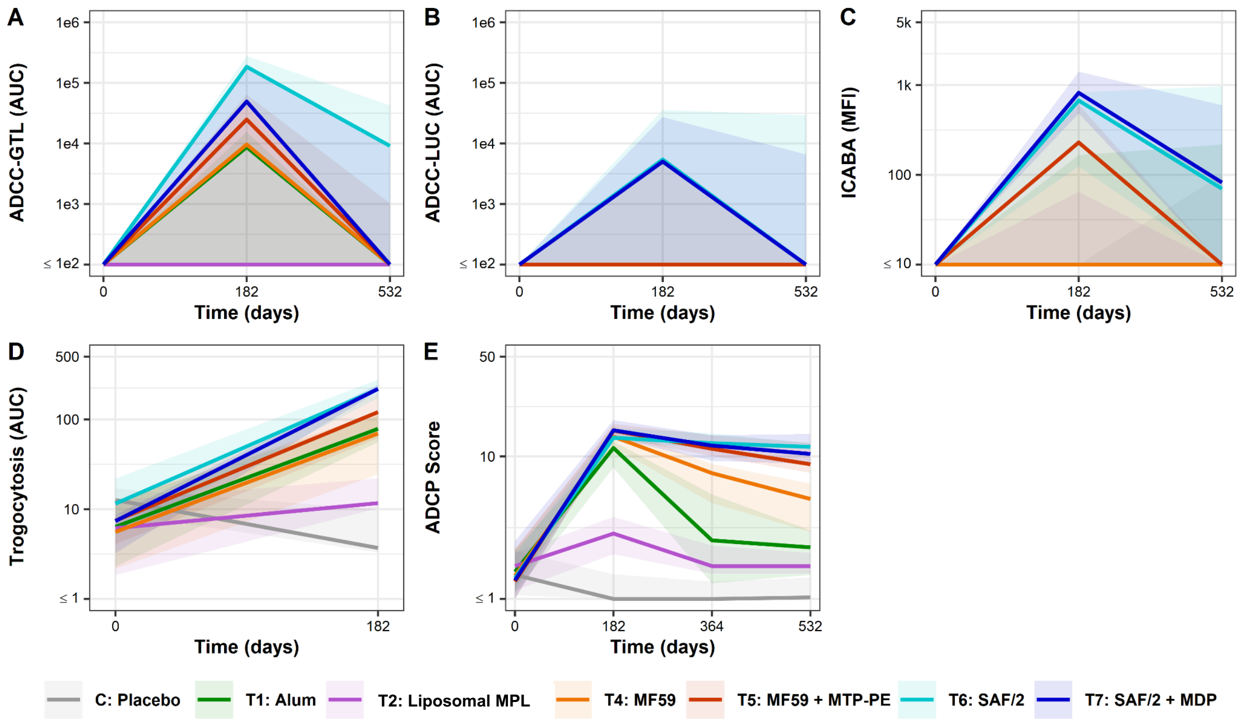

Supplemental Figure 1. Longitudinal profile of SF2-specific antibody functions. Antibody dependent cellular cytotoxicity GranToxiLux (ADCC-GTL) area under the curve (AUC) (**A**), Antibody dependent cellular cytotoxicity Luciferase (ADCC-LUC) area under the curve (AUC) (**B**), Infected Cell Antibody Binding Activity (ICABA) in mean/median fluorescence intensity (MFI) (**C**), Trogocytosis AUC (**D**), and Antibody-dependent cellular phagocytosis (ADCP) score (**E**) over time. Solid lines depict median response over time, and the shaded regions represent the interquartile range (25th percentile to 75th percentile), colored by treatment arm.

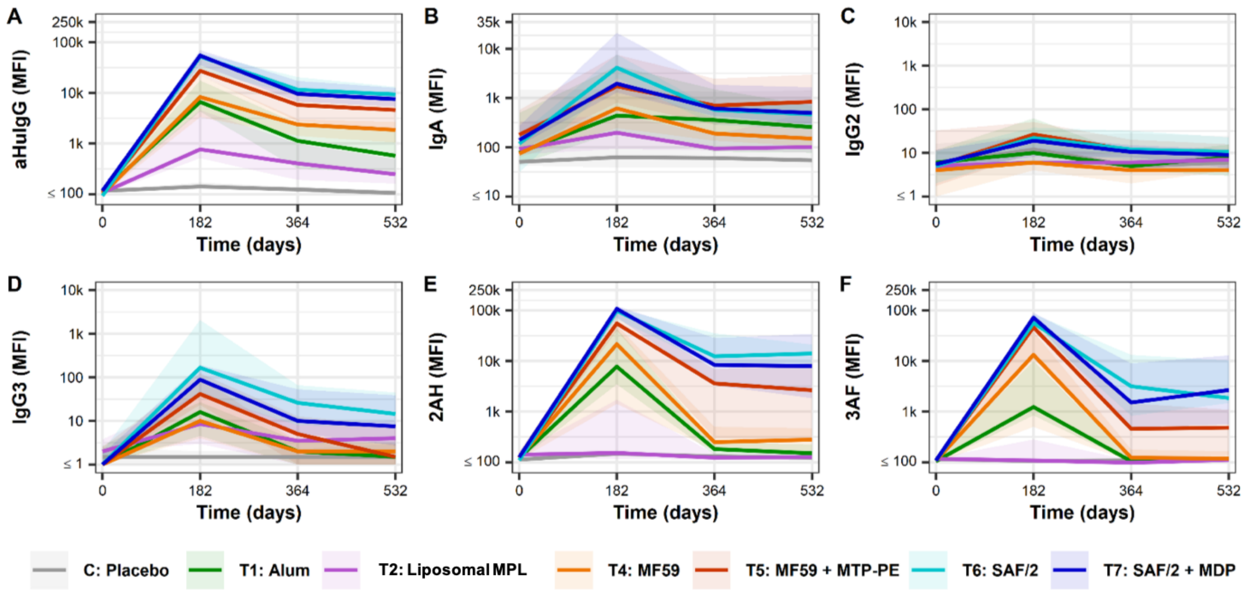

Supplemental Figure 2. Longitudinal profile of SF-2-specific antibodies. Fc Array characterization of antibodies raised against SF-2 gp120 across subclass (**A-D**) and Fcγ receptor binding (**E-F**) over time. Solid lines depict median response over time, and the shaded regions represent the interquartile range (25th percentile to 75th percentile), colored by treatment arm.

Supplemental Table 1: Antigens used in BAMA/BAMA-AI and Fc Array Assays.

V1V2 gp70/Tags	gp140	gp120	V3 and Others
**BAMA/BAMA-AI**			
gp70-001428.2.42 V1V2	A1.con.env03 140 CF	A244 D11gp120_avi	B.MN V3 gp70
gp70-191084_B7 V1V2	AE.01.con_env03 gp140CF_avi	B.63521_D11gp120/293F	Bio-V3.A
gp70-700010058 V1V2	BF1266_gp140C.avi/293F	BJOX002_D11gp120.avi/293F	Bio-V3.B
gp70-7060101641 V1V2	Con S gp140 CFI	BORI_D11gp120.avi/293F	Bio-V3.C
gp70-96ZM651.02 V1v2	RHPA4259_C7.gp140C.avi	CNE20_D11gp120.avi/293F	Bio-V3.CRF1
gp70-BF1266_431a_V1V2	SC42261_gp140.avi/293F	TT31P.2792_D11gp120.avi/293F	Bio-V3.CRF2
gp70-BJOX002000.03.2 V1V2	WITO4160.gp140C.avi	TV1c8_D11gp120.avi/293F	Bio-V3.M
gp70-C2101.c01_V1V2	00MSA 4076 gp140	1086C_D7gp120.avi/293F	HxB2 new 8b core 6x His
gp70-CAP210.2.00.E8 V1V2	1086C gp140C_avi	254008_D11gp120.avi/293F	RSC3
gp70-CM244.ec1 V1V2		51802_D11gp120.avi/293F	YU2 gp120 old core
gp70-RHPA4259.7 V1V2		96ZM651.D11gp120.avi	YU2 gp120 WT
gp70-TT31P.2F10.2792 V1V2			HxB2 new 8b core 1420R
gp70-TV1.21 V1V2			RSC3 delta371
gp70_B.CaseA_V1_V2			RSC3 delta371/P363N
AE.A244 V1V2 tags			YU2 gp120 1420R
AE.A244 V1V2 Tags/293F			YU2 gp120 old core D368R
C.1086C_V1_V2 Tags			
**Fc array**			
CaseA2 gp70 V1V2	B.6240 gp140 gp140C/293F	620345_D11 gp120.avi/293F	
Ce704010042_2E5 gp70-V1V2	C3347_11 gp140C.avi	Q769_D11 gp120.avi/293F	
7060101641A7 gp70-V1V1	CNE5.gp140C.avi.293F	62357_D11 gp120.avi/293F	
REJO4541.67 gp70-V1V2	QH0515.gp140C.avi	BJOX028_D11 gp120.avi/293F	
gp70 scaffold (control) WT		C.089_D11 gp120/293F	
		gp120 JRCSF	
		gp120 PVO	
		CAP45_D11 gp120.avi/293F	
**BAMA/BAMA-AI and Fc array**			
gp70-62357.14 V1V2	C.CH505TF_gp140/293F	B.6240_D11gp120/293F	
gp70-Ce1086_B2 V1V2	9004S.gp140C.avi	SF2 gp120	
