# Supplementary material for: Impact of adjuvants on the biophysical and functional characteristics of HIV vaccine-elicited antibodies in humans
Source: NPJ Vaccines. 2022 Aug 4;7:90. doi: 10.1038/s41541-022-00514-9 (PMC9352797; doi:10.1038/s41541-022-00514-9)
Supplement: Supplementary file 2 — REPORTING SUMMARY [file 41541_2022_514_MOESM2_ESM.pdf]

nature portfolio

Corresponding author(s): Margaret E. Ackerman

Last updated by author(s): May 26, 2022

Reporting Summary

Nature Portfolio wishes to improve the reproducibility of the work that we publish. This form provides structure for consistency and transparency in reporting. For further information on Nature Portfolio policies, see our Editorial Policies and the Editorial Policy Checklist.

Statistics

For all statistical analyses, confirm that the following items are present in the figure legend, table legend, main text, or Methods section.

n/a	Confirmed	
☐	☒	The exact sample size (*n*) for each experimental group/condition, given as a discrete number and unit of measurement
☐	☒	A statement on whether measurements were taken from distinct samples or whether the same sample was measured repeatedly
☐	☒	The statistical test(s) used AND whether they are one- or two-sided<br>*Only common tests should be described solely by name; describe more complex techniques in the Methods section.*
☐	☒	A description of all covariates tested
☐	☒	A description of any assumptions or corrections, such as tests of normality and adjustment for multiple comparisons
☐	☒	A full description of the statistical parameters including central tendency (e.g. means) or other basic estimates (e.g. regression coefficient) AND variation (e.g. standard deviation) or associated estimates of uncertainty (e.g. confidence intervals)
☐	☒	For null hypothesis testing, the test statistic (e.g. *F*, *t*, *r*) with confidence intervals, effect sizes, degrees of freedom and *P* value noted<br>*Give P values as exact values whenever suitable.*
☒	☐	For Bayesian analysis, information on the choice of priors and Markov chain Monte Carlo settings
☒	☐	For hierarchical and complex designs, identification of the appropriate level for tests and full reporting of outcomes
☐	☒	Estimates of effect sizes (e.g. Cohen's *d*, Pearson's *r*), indicating how they were calculated

Our web collection on statistics for biologists contains articles on many of the points above.

Software and code

Policy information about availability of computer code

Data collection	Bioplex manager and luminex software and various flow cytometer softwares were used across assays. Methods section references full information.
Data analysis	R software, packages bnstruct, glmnet, WGCNA, goodSamplesGenes, ggraph, and hearmap3 were used. Original references for each cited within manuscript.

For manuscripts utilizing custom algorithms or software that are central to the research but not yet described in published literature, software must be made available to editors and reviewers. We strongly encourage code deposition in a community repository (e.g. GitHub). See the Nature Portfolio guidelines for submitting code & software for further information.

Data

Policy information about availability of data

All manuscripts must include a data availability statement. This statement should provide the following information, where applicable:

- Accession codes, unique identifiers, or web links for publicly available datasets
- A description of any restrictions on data availability
- For clinical datasets or third party data, please ensure that the statement adheres to our policy

Data is available from study authors upon reasonable request.

Human research participants

Policy information about studies involving human research participants and Sex and Gender in Research.

Reporting on sex and gender	This information has not been reported. The study was not powered appropriately for inclusion
Population characteristics	This information was not provided for this study.
Recruitment	Ages Eligible for Study: 18 Years to 60 Years (Adult)<br>Sexes Eligible for Study: All<br>Accepts Healthy Volunteers: Yes<br>Criteria<br>Inclusion Criteria<br>Subjects must have:<br>HIV negativity by ELISA.<br>Normal history and physical exam.<br>CD4 count >= 400 cells/mm3.<br>Lower risk sexual behavior.<br>Normal urine dipstick with esterase and nitrite.<br>PER AMENDMENT 3/6/96:<br>Extension study -<br>Consenting Protocol 015 volunteers who have received four immunizations.<br>Exclusion Criteria<br>Co-existing Condition:<br>Subjects with the following symptoms or conditions are excluded:<br>Hepatitis B surface antigen.<br>Active syphilis. NOTE:Subjects for whom serology is documented to be a false positive or due to a remote (> 6 months) treated infection are eligible.<br>Active tuberculosis. NOTE:Subjects with a positive PPD and normal chest x-ray showing no evidence of TB and not requiring isoniazid therapy are eligible.<br>Medical or psychiatric condition or occupational responsibilities that would preclude compliance.<br>Subjects with the following prior conditions are excluded:<br>History of immunodeficiency, chronic illness, or autoimmune disease.<br>History of anaphylaxis or other serious adverse reactions to vaccines.<br>PER AMENDMENT 3/6/96: Extension study -<br>History of eczema or allergic-type reactions to vaccine in Protocol 015.<br>Prior Medication:<br>Excluded:<br>Live attenuated vaccines within 60 days prior to study entry. (NOTE: Medically indicated subunit or killed vaccines, such as influenza or pneumococcal, are allowed but should be given at least 2 weeks prior to HIV immunizations.)<br>Experimental agents within 30 days prior to study entry.<br>Prior HIV vaccines.<br>PER AMENDMENT 3/6/96: Extension study -<br>Use of systemic steroids in the past month.<br>Prior Treatment:<br>Excluded:<br>Blood products or immunoglobulin within 6 months prior to study entry. Higher risk behavior for HIV infection (as determined by screening questionnaire), including history of injection drug use within the last 12 months and higher or intermediate risk sexual behavior.
Ethics oversight	Duke University

Note that full information on the approval of the study protocol must also be provided in the manuscript.

Field-specific reporting

Please select the one below that is the best fit for your research. If you are not sure, read the appropriate sections before making your selection.

☒ Life sciences ☐ Behavioural & social sciences ☐ Ecological, evolutionary & environmental sciences

For a reference copy of the document with all sections, see nature.com/documents/nr-reporting-summary-flat.pdf

Life sciences study design

All studies must disclose on these points even when the disclosure is negative.

Sample size	All available samples for the clinical trial were tested excluded one adjuvant group for which permission for study was not recieved.

Data exclusions	Data from all tested subjects was included in analysis.
Replication	Data analysis was replicated using repeated cross-validation. Experimental replicates were determined per assay on the basis of prior work, and details are provided in references.
Randomization	Randomization was performed at the time of the clinical trial.
Blinding	Samples were blinded at the time of assay.

Reporting for specific materials, systems and methods

We require information from authors about some types of materials, experimental systems and methods used in many studies. Here, indicate whether each material, system or method listed is relevant to your study. If you are not sure if a list item applies to your research, read the appropriate section before selecting a response.

Materials & experimental systems

n/a	Involved in the study
☐	☒ Antibodies
☐	☒ Eukaryotic cell lines
☒	☐ Palaeontology and archaeology
☒	☐ Animals and other organisms
☐	☒ Clinical data
☒	☐ Dual use research of concern

Methods

n/a	Involved in the study
☒	☐ ChIP-seq
☒	☐ Flow cytometry
☒	☐ MRI-based neuroimaging

Antibodies

Antibodies used	a- IgG Southern Biotech 1030-09<br>a-IgG2 Southern Biotech 9070-09<br>a-IgG3 Southern Biotech 9210-09<br>a-IgA Southern Biotech 2050-09
Validation	The above are widely used secondary antibodies with well-established recognition profiles as described by their manufacturer, available at: https://www.southernbiotech.com

Eukaryotic cell lines

Policy information about cell lines and Sex and Gender in Research

Cell line source(s)	THP-1 ATCC TIB-202<br>CEM.NKRCCR5 National Institute of Allergy and Infectious Diseases (NIAID) Reagent Repository<br>EGFP-CEM-Nkr National Institute of Allergy and Infectious Diseases (NIAID) Reagent Repository
Authentication	Cells were procured from reliable sources such as ATCC.
Mycoplasma contamination	Mycoplasma testing status is not known.
Commonly misidentified lines<br>(See ICLAC register)	*Name any commonly misidentified cell lines used in the study and provide a rationale for their use.*

Clinical data

Policy information about clinical studies

All manuscripts should comply with the ICMJE guidelines for publication of clinical research and a completed CONSORT checklist must be included with all submissions.

Clinical trial registration	NCT00001042
Study protocol	Available from Protocol Chair McElrath J.
Data collection	samples:<br>completed in 1996<br>United States, Missouri<br>St. Louis Univ. School of Medicine AVEG<br>Saint Louis, Missouri, United States, 63104<br>United States, New York<br>Univ. of Rochester AVEG

Rochester, New York, United States, 14642
United States, Washington
UW - Seattle AVEG
Seattle, Washington, United States, 98144

Experimental Data:
2015-2022 at Duke University, Dartmouth College, and University of Maryland

Outcomes	This study analyzed meaures that were not primary or secondary outcomes in the original trial design.
